# Carvone derived cannabidiol enantiomers as novel anticonvulsants

**DOI:** 10.1038/s41386-025-02220-1

**Published:** 2025-09-24

**Authors:** Rochelle M. Hines, April Contreras, Adriana Carrillo, Alexandra Paton, Antonio J. Tenorio, William A. Maio, Dustin J. Hines

**Affiliations:** 1 Department of Psychology, Psychological & Brain Sciences, Interdisciplinary Neuroscience Program, University of Nevada Las Vegas, Las Vegas, NV USA; 2 Department of Chemistry and Biochemistry, New Mexico State University, Las Cruces, NM USA

**Keywords:** Target validation, Developmental disorders

## Abstract

Developmental epilepsy syndromes are characterized by recurrent seizures and developmental delays. Current anticonvulsants target γ-aminobutyric acid type A receptor signaling to decrease neuronal excitability, however, there are adverse effects for the developing brain, and many patients are refractory. The major non-psychotropic phytocannabinoid cannabidiol (CBD) has emerged as an anti-seizure medication effective in select developmental epilepsy syndromes, but its overall applicability in treating seizure disorders is limited. In the present study, we characterize a small library of non-Cannabis carvone derived CBD (+) enantiomers, with the larger goal of identifying novel therapeutics for developmental epilepsy syndromes. EEG based structure activity relationship assessment supports that elongated alkyl chains increase the potency of the congeners, with (+)-CBD-oct displaying effects on both δ and θ frequency bands. Pre-treatment with (+)-CBD-oct promotes seizure resilience in both wildtype mice and the *Gabra2*-1 model of developmental epilepsy by influencing seizure characteristics, and reduces mortality. 5 days of (+)-CBD-oct oral gavage in wildtype and *Gabra2*-1 mice during postnatal development normalizes the aberrant dendritic spine phenotype of *Gabra2*-1 mice. These findings advance the development of novel anticonvulsants by validating an influence of alkyl chain length of synthetic CBD congeners.

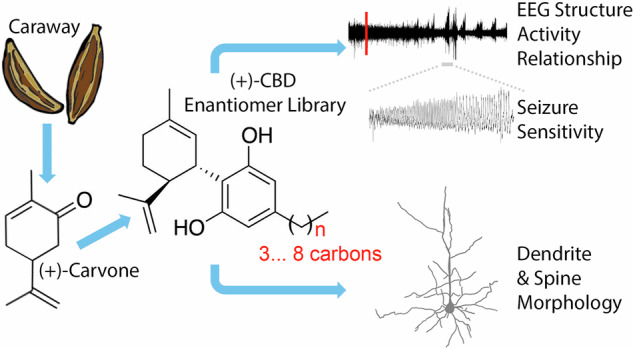

## Introduction

Developmental epilepsy syndromes are characterized by recurrent seizures, frequently accompanied by developmental delays. Children with these rare genetic syndromes, such as Dravet and Lennox-Gastaut, present with variable seizure subtypes, become resistant to anti-seizure medications, and often experience life-threatening breakthrough seizures following intense regiments of pharmacological and non-pharmacological therapies [1–4]. Beyond these examples, a large number of neurodevelopmental disorders caused by both common [5, 6] and rare [7–11] mutations have a high incidence of epilepsy that can be encephalopathic and exacerbate intellectual disability [12–14]. One common core characteristic of developmental epilepsy syndromes is a lack of inhibitory control of principal pyramidal cell excitability [15–17]. Benzodiazepines and other sedative-hypnotics, which allosterically modulate the ionotropic γ-aminobutyric acid type A receptor (GABA_A_R), are the primary antiepileptic used clinically [18–21]. Seizures are typically managed with a combination of 2 or more benzodiazepines with supplemental non-pharmacological treatments. Chronic benzodiazepine treatment leads to rapid tolerance, and can result in physical dependence, as well as the development of refractory epilepsy [22]. Additional adverse effects of these anti-seizure medications in children include increased somnolence, gastrointestinal dysfunction, respiratory depression, and cognitive deficits [23, 24].

Cannabidiol (CBD), one of the major phytocannabinoids found in the *Cannabis sativa* plant, demonstrates clinical utility in the treatment of seizures [25–29]. Unlike Δ^9^ tetrahydrocannabinol (THC), CBD does not produce psychotropic effects [30, 31]. Currently, Epidiolex is the only FDA approved purified CBD solution that can be prescribed to infants and children with Lennox-Gastaut syndrome, Dravet syndrome, or tuberous sclerosis complex. While Epidiolex alone or in combination with other anticonvulsants may help prevent and reduce seizures, chronic treatment to maintain resistance to seizure has been associated with adverse effects [32, 33]. The clinical applicability of Epidiolex beyond its current FDA-approved indications remains unestablished, particularly for other neurodevelopmental disorders characterized by a high incidence of epilepsy. There has been limited investigation of CBD analogs and other cannabimimetics for the potential discovery of both generalizable and personalized antiepileptic therapies [34–37]. Fully synthetic, non-natural CBD derivatives offer a compelling alternative to semi-synthetic analogs derived from plant-extracted CBD [37, 38] through elimination of psychoactive THC contamination, and by providing the flexibility to precisely modify pharmacophoric features to fine-tune therapeutic activity.

The development of non-natural CBD derivatives to treat seizures relies on an understanding of natural cannabinoid structure and the range of receptor targets that have been identified for CBD. There are over 100 unique phytocannabinoids produced by *Cannabis sativa*, which differentially modulate CB receptors (CB_x_Rs), including THC, CBD, cannabidivarin (CBDV), and cannabidiphorol (CBDP) [39]. Pytocannabinoids share a resorcinol core and cyclohexane ring system and naturally occur as (–) enantiomers, but vary in the length of the resorcinyl side chain with THC and CBD having 5 carbons, CBDV having 3, and CBDP having 7 [40–42]. Structure activity relationship (SAR) studies in vitro and in vivo have identified the length of the alkyl side chain as a critical pharmacophore, changing the conformational mobility of cannabinoids [43].

THC facilitates psychotropic effects via partial agonism at CB_1_Rs and CB_2_Rs. Increasing alkyl side chain length increases THC binding affinity for the CB_1_R up to 29-fold [41, 44]. By contrast, the (+) enantiomer of THC (( + )-THC) has very low affinity for CB_1_Rs, and is minimally psychoactive [45, 46]. CBD is also a partial agonist for CB_2_Rs, however, its actions on CB_1_Rs are less clear [47]. CBD has low affinity for the orthosteric site of CB_1_Rs [48] and has even been shown to have antagonistic effects at CB_1_Rs [49]. Recent studies based on CBD-bound crystal structures suggest that CBD is likely a negative allosteric modulator of THC and endogenous cannabinoid agonism at CB_1_Rs [50]. Presynaptic expression of CB_1_Rs regulates glutamatergic and GABAergic neurotransmitter release, and CBD is known to interact directly at other receptor types [51–53]. Of relevance to CBD’s utility as an anti-seizure medication is preclinical evidence that CBD enhances GABA evoked current at α2-containing GABA_A_ receptors [54], acts as a partial agonist or positive allosteric modulator at 5HT_1A_Rs [55–57], acts as an agonist at subtypes of Transient Receptor Potential (TRP) channels [58, 59], and also as an antagonist of the G protein coupled receptor GPR55 [60, 61]. ( + )-CBD has also been shown to activate sphingosine-1 phosphate (S1P) receptors, S1P_1_ and S1P_3_ in a stereoselective manner [62]. While alkyl chain length enhances the affinity of phytocannabinoids for CB_1_Rs [43], an inverse relationship has been suggested for TRP channel activity [63, 64], and the impact on other targets remains uncharacterized. The broad range of cannabinoid targets, both canonical and non-cannabinoid receptors, further supports the investigation of derivatives that may be useful in strengthening or honing CBDs antiepileptic action.

In the present study, we produce a library of (+)-CBD derivatives from enantiopure (+)-carvone, which is an extract from the seeds of the caraway plant, *Carum carvi* [65]. We hypothesize that (+)-enantiomers will have minimal activity at CB_1_Rs due to stereoselectivity thereby minimizing possible psychoactive effects, while varying the lengths of alkyl chains will tune (+)-CBD congeners for non-canonical receptor targets. We find that altering the alkyl chain length of (+)-CBD enantiomers produces distinct effects on cortical activity and EEG frequency bands in mice, guiding the selection of (+)-CBD-oct as a lead candidate. Open field testing reveals that (+)-CBD-oct lacks acute sedative effects. In both chemical and genetic seizure models, including the *Gabra2*-1 chimera, ( + )-CBD-oct confers significant resistance to kainic acid-induced mortality. Repeated treatment from P5–P10 normalizes aberrant spine morphology in *Gabra2*-1 pups without affecting wildtype littermates. These findings highlight the therapeutic potential of elongated alkyl chain (+)-CBD derivatives for treating developmental epilepsy syndromes and support further development of this compound class.

## Materials and methods

### Library synthesis

Each of the enantiomer CBD derivatives used in this study were prepared from (+)-carvone in five synthetic steps according to our previously reported protocol [38, 65]. The reactions employed were performed in flame-dried glassware under an atmosphere of dry nitrogen. Reaction solvents were purified before use in a Glass Contour Solvent Purification System and all reagents were purchased from Sigma-Aldrich and used as received. Each derivative was purified on silica gel using a Teledyne CombiFlash chromatography system before use. Additional details of the library synthesis, including structural confirmation data, are included in the supplementary materials.

### Animals and sex as a biological variable

Animals were cared for according to the NIH Guide for the Care and Use of Laboratory Animals, and protocols were approved by the Institutional Animal Care and Use Committee (IACUC) of the University of Nevada, Las Vegas. The reported studies were designed based on the ARRIVE guidelines (Animal Research: Reporting of In Vivo Experiments [66]). Mice were group housed with a 12 h light-dark cycle with constant access to food and water. Cohorts of juvenile and adult C57Bl6 and *Gabra2*-1 (heterozygous and homozygous) littermates were used in the present studies, and cohorts were tested at the same time of day on multiple days as needed. Adult animal studies examined male mice because male animals exhibit less variability in the baseline EEG and in the response to chemical seizure induction. It is unknown whether the findings are relevant for female mice. Studies in pups examined male and female mice, and similar findings were found for both sexes.

### Pharmacology

CBD enantiomers were prepared by dissolving in sesame oil and administered with ddH_2_O via oral gavage (p.o.) according to the weight of the animal to achieve a 20 mg*kg^-1^ dose. The dose of 20 mg*kg^-1^ was selected to be at the low end of the established efficacy range for (-)-CBD in preclinical models, as well as in translational alignment with clinically approved dosing in patients with developmental epilepsies. In rodent studies, (-)-CBD demonstrates anti-seizure effects across a variety of models, within a dose range of 10–100 mg*kg^-1^, with 20–50 mg*kg^-1^ frequently used as a non-sedating, behaviorally tolerable range [67, 68]. Clinically, Epidiolex is approved at doses up to 25 mg*kg^-1^/day for treatment-resistant epilepsies such as Dravet syndrome, Lennox-Gastaut syndrome, and tuberous sclerosis complex [68]. The vehicle consisted of ddH_2_O alone. Kainate (Hello Bio) was administered intraperitoneally at a 20 mg*kg^-1^ dose.

### Electroencephalography (EEG)

EEG and electromyography (EMG) recordings were performed as previously described [10, 69] with additional details provided here. Implantation: Anesthesia was induced and maintained using isoflurane. A small incision was made on the scalp exposing the skull. Four insulated wire electrodes were placed and screwed as follows: two extradural cortical electrodes were inserted bilaterally in the frontal areas and the two others were inserted bilaterally in the parietal/occipital areas. For implantation of EMG, two insulated wire electrodes were inserted bilaterally into the nuchal muscle. Electrodes connected to a microconnector (Pinnacle Technology) were secured at the surface of the skull with dental acrylic. Following implantation animals were given a postoperative injection of saline for hydration and singly housed. Mice were given 1 week to recover before experimentation. Recording: The recording apparatus consisted of a head mount that connected to a pre-amplifier, commutator, digitizer, and a computer with Sirenia Acquisition (Pinnacle Technologies, Lawrence, KS). The Pinnacle EEG/EMG system is engineered to be artifact free, with amplification and filtering at the head by the preamplifier, and further by the data conditioning and acquisition system. Data were acquired with a sampling rate of 1 kHz, low pass 0.1 Hz, high pass 100 Hz. Mice were individually placed in a clear plexiglass cylinder with cob bedding and the pre-amplifier was plugged-in to the head mount implant for at least 1 h to habituate to the recording apparatus and tether. Recordings were taken of 1 h of baseline activity, beginning 2–4 h into the dark phase of the light dark cycle. During baseline recordings, animals were visually monitored under red light and sleep was suppressed with the introduction of novel objects in order to help establish a homogenous waking EEG baseline. After baseline, mice were administered (+)-CBD derivatives (red line; 20 mg*kg^-1^) via oral gavage (p.o.) followed by an additional 60 min of data collection. We also performed experiments to compare to p.o. vehicle as an additional control and found no difference between baseline and p.o. vehicle (Supplementary Fig. 2). For kainate experiments, ( + )-CBD-oct was administered p.o. 30 min prior to i.p. injection of 20 mg*kg^-1^ kainate (yellow line). Data collection continued for up to 180 min or until the mouse expired from seizure. A seizure event was defined as a sudden onset of high amplitude activity greater than 2.5 times the standard deviation of the baseline EEG recording, with the duration of activity required to exceed 5 s to meet criteria. When an animal had 2 events separated by <2 s the animal was defined as entering status epilepticus (SE). In animals having only a single event, time to SE was censored at the maximum duration of the experiment (180 min). Acquired EEG data was exported from Sirenia Acquisition and processed using Sleep Sign for animal (KISSEI COMTEC CO, Nagano, Japan) to generate cumulative fast Fourier transform (cFFT) data, and to allow epoch screening for artifacts. No movement artifacts were observed. MATLAB (MATLAB 2019) was used for further analysis of spectral frequencies and to generate spectrograms. Frequency bands were defined as δ (0.4–4.0 Hz), θ (4.5–8.0 Hz), α (8.5–13.0 Hz), β (13.5–30 Hz), and γ (30.5–100.0 Hz). Data were normalized to baseline δ. Results were quantified using SleepSign, Clampfit, and MATLAB.

### Behavioral assessment of seizures

The Racine scale modified for mice was used to behaviorally score seizures, with 0 = normal behavior/no change; 1 = behavioral arrest, shivering; 2 = head nodding, Straub tail; 3 = forelimb clonus, champing; 4 = forelimb clonus with rearing; 5 = generalized tonic clonic, wild jumping; 6 = expired from seizure. Survival was monitored for up to 180 min or until the mouse expired from seizure, with surviving animals censored at 180 min.

### Open field behavior

Mice were habituated to a testing room for 1 h before oral gavage of either vehicle (ddH_2_O) or (+)-CBD-oct (20 mg*kg^-1^). Mice were then returned to their home cage for 30 min before being placed in the open field apparatus for 60 min. Open field behavior of mice was assessed using ANY-Maze video tracking software from video recordings taken from above. Paths and heat maps plotting movement and immobile episodes were made using MATLAB.

### Histology

For analysis of neuronal morphology and dendritic spines, brains were drop fixed in Golgi-Cox solution and stored in the dark for 10 days. Brains were then transferred to 30% sucrose solution and stored in the dark for an additional 3 days. Brain slices were prepared at a thickness of 200 µm using a Precisionary Instruments Compresstome and mounted onto 2% gelatinized slides to be developed by a modified Golgi Cox technique [70]. Brain slices were embedded in Permount and coverslipped.

### Statistics

All graphs were created using SigmaPlot, with line graphs and bar graphs plotted as mean and standard error, and box plots plotted as median, first and third quartile, and range. After normality testing (Shapiro-Wilk or Kolmogorov-Smirnov) and testing for equal variance (Brown-Forsyth), analysis was performed using parametric tests including *t*-test, one-way, or repeated measures ANOVA with Bonferroni post hoc analysis when appropriate. Exact *p*-values are shown in figures when the threshold for significance ( < 0.05) is met, in some cases, “n.s.” denotes non-significant results for clarity. Although the kainite induced seizure EEG recording experiments were conducted with small group sizes (*n* = 5 per group), the observed effects were statistically significant and biologically robust, justifying the conclusions despite limited statistical power. This approach aligns with ethical guidelines to reduce animal morbidity and overall use, while prioritizing data quality and feasibility in high-burden assays. Kainate seizure survival data were plotted as Kaplan-Meier curves, followed by Log Rank analysis.

## Results

### Synthesis of (+)-CBD derivatives using a novel synthetic method with (+)-carvone as starting material

**Fig. 1:**
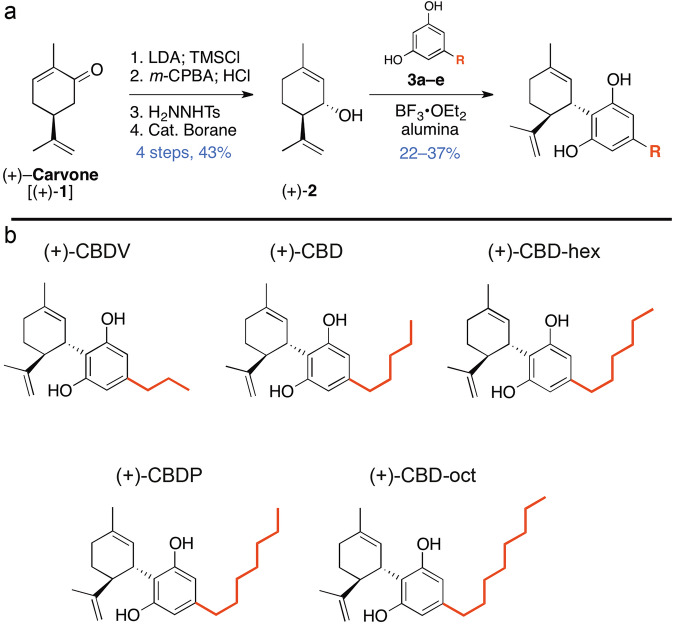
Schemes of synthesis of enantiomer-CBD derivatives and final structures. **a** Conversion of (+)-carvone into (-)-isopiperitenol in the synthesis of (+)-CBD. This process relies on the one-pot borane reduction / thermal rearrangement of an α,β-unsaturated hydrazone. **b** By uniting synthetic (+)-isopiperitenol with olivetol derivatives (Ar-H) under buffered Lewis acid conditions, we prepared a library of non-natural, enantiomer-CBD derivatives, each tailored with a different alkyl chain. While the derivatives with an odd number of alkyl carbons are known in their natural form, compounds featuring an even number of alkyl carbons have never been previously reported in either enantiomer form.

A novel, inexpensive, and stereoselective method was developed to design a small library of synthetic (+)-CBD derivatives using (+)-carvone as a starting material [38] (Fig. 1a). ( + )-carvone derived (+)-CBD molecules were designed with increasing numbered alkyl chains from three ((+)-CBDV) to eight ((+)-CBD-oct) carbon atoms in length (Fig. 1b). Treatment of (+)-carvone with in situ prepared Lithium Diisopropylamide (LDA), followed by the addition of TMSCl allowed access to the corresponding silyl enol ether, which was directly treated with *m*-chloroperoxybenzoic acid (*m*-CPBA) to afford α-hydroxy carvone as a mixture of stereoisomers. Once purified, the major diastereomer was reacted with tosylhydrazide and the corresponding hydrazone was treated with catecholborane to afford (+)-isopiperitenol in good yield after concomitant hydride reduction and alkene transposition [43% overall yield (four steps) from (+)-carvone]. In order to generate (+)-CBD derivatives, ( + )-isopiperitenol was then separately united with olivetol derivatives using borontrifluoride diethyl etherate (BF_3_•OEt_2_) as catalyst. After silica gel purification, isolated yields for this final step ranged from 22-37%. The library examined in the present studies contains the enantiomers (+)-CBDV, ( + )-CBD, ( + )-CBDP, and non-natural (+)-CBD-hex, and (+)-CBD-oct (Fig. 1b) derivatives, well suited to examine the influence of stereoselectivity and alkyl chain length on the action of CBD derivatives.

### Synthetic (+)-CBD derivatives produce distinct effects on electroencephalographic frequency band activity

**Fig. 2:**
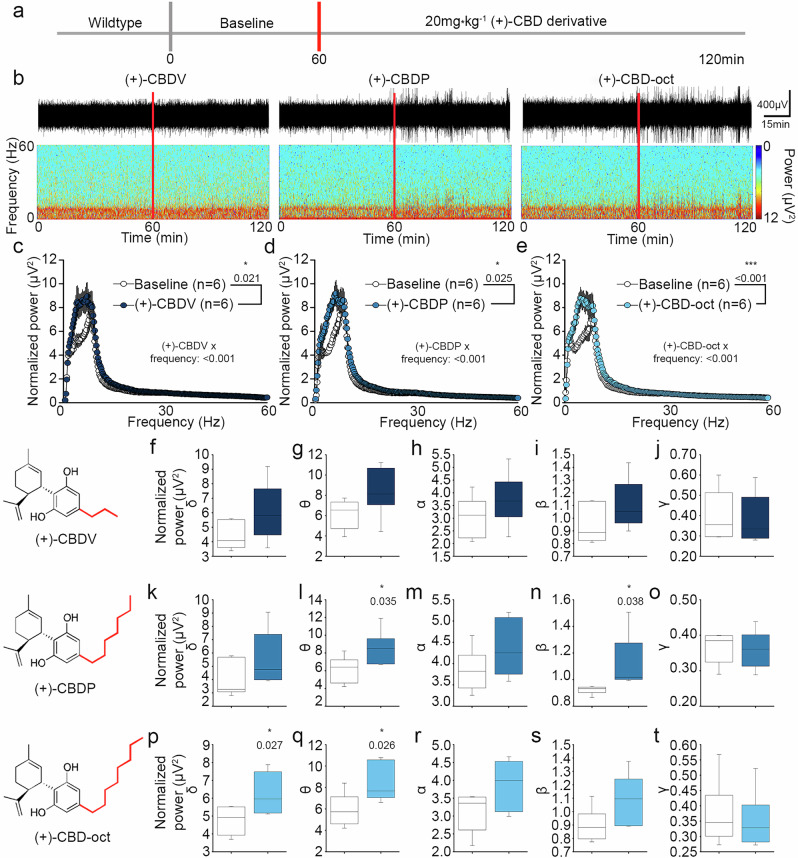
Structure activity relationship (SAR) study of (+)-enantiomer-CBD derivatives using EEG recordings. **a** Timeline of SAR, red lines denote administration of (+)-CBD derivative (20 mg*kg^-1^ p.o.). **b** Representative traces (top) and spectrograms (bottom) of EEG recordings following 20 mg*kg^-1^ p.o. with (+)-CBD derivatives. **c**–**e** Fast Fourier transform analysis of the EEG signal comparing (+)-CBDV (Baseline: 0.998 ± 0.0419, ( + )-CBDV: 1.196 ± 0.0419), ( + )-CBDP (Baseline: 0.996 ± 0.0463, ( + )-CBDP: 1.204 ± 0.0463), and (+)-CBD-oct (Baseline: 0.984 ± 0.0210, ( + )-CBD-oct: 1.188 ± 0.0210), to vehicle baseline. **f**–**j** Spectral analysis of (+)-CBDV (All bands n.s.). **k**–**o**. Spectral analysis of (+)-CBDP (θ Baseline: 6.149 ± 1.468, β (+)-CBDP: 0.923 ± 0.0303). **p**–**t** Spectral analysis of (+)-oct compared to baseline (δ Baseline: 4.764 ± 0.822, δ (+)-CBD-oct: 6.242 ± 1.137; θ Baseline: 5.918 ± 1.780, θ (+)-oct: 8.407 ± 1.780). All values listed are mean (or LS mean) ± standard error, *p* values from Two Way Repeated Measures ANOVA, with Bonferroni post hoc; or t-test. Line graphs are plotted as mean and standard error, and box plots are plotted as median, first and third quartile, and range.

To determine the effects of novel (+)-CBD derivatives on circuit-level activity, we conducted SAR studies using EEG in freely moving mice. Animals received 20 mg*kg^-1^ of a (+)-CBD derivative via oral gavage (p.o.) and EEG activity was recorded for 60 min (Fig. 2a). EEG traces and spectrograms of a subset of (+)-CBD derivatives tested suggest deviation from baseline EEG activity in the hour following oral gavage (Fig. 2b), including (+)-CBDV (20 mg*kg^-1^), ( + )-CBDP (20 mg*kg^-1^), and (+)-CBD-oct (20 mg*kg^-1^) derivatives. To further identify the specific frequency bands driving the deviation from baseline, a cumulative fast Fourier transform (cFFT) analysis was conducted (Fig. 2c–e). Plotted cFFTs and subsequent analysis show that (+)-CBDV, ( + )-CBDP, and (+)-CBD-oct significantly increase power compared to baseline (Fig. 2c–e). The data was then parsed into frequency bands (0.4–4 Hz–δ; 4–8 Hz–θ, 8-13 Hz α, 13-30 Hz– β, 30–100 Hz–γ) and spectral analysis reveals that (+)-CBDV does not significantly increase any specific frequency band power compared to baseline, with only trending increases across δ, θ, α, and β frequency bands (Fig. 2f–j). The (+)-CBDP derivative significantly increases θ and β power (Fig. 2k–o), and (+)-CBD-oct specifically increases both δ and θ frequency band power (Fig. 2p–t). ( + )-CBD and (+)-CBD-hex produce less robust effects on EEG (Supplementary Fig. 1) with minimal changes in power compared to baseline following oral gavage administration of (+)-CBD (Supplementary Fig. 1a, b) and (+)-CBD-hex (Supplementary Fig. 1a, c). Spectral analysis reveals no significant power changes in any parsed frequency band compared to baseline for (+)-CBD (Supplementary Fig. 1d–h) or (+)-CBD-hex (Supplementary Fig. 1i–m). These findings functionally support the notion that modifications to alkyl chain length of synthetic carvone-derived (+)-CBD-derivatives produce variable changes in EEG frequency power. ( + )-CBD-oct was then selected as the lead candidate for additional studies due to its robust effects on low frequency bands of δ and θ power compared to baseline, indicating that it may reduce epileptic activity. Taken together, the SAR studies demonstrate synthetic carvone-derived (+)-CBD enantiomers with varying alkyl chain length have nuanced effects on circuit-level activity, and functionally substantiate the proposed impact of elongated alkyl chains on CBD derivative action.

### ( + )-CBD-oct does not reduce exploration in the open field

**Fig. 3:**
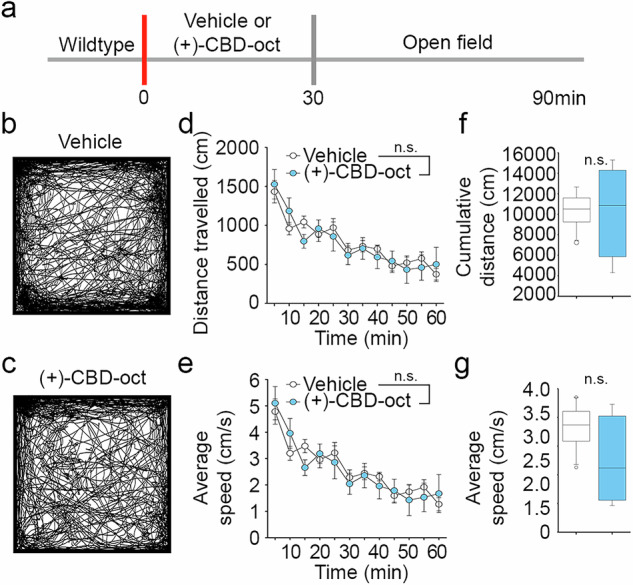
Enantiomer-CBD derivative (+)-CBD-oct is does not produce sedative effects at a therapeutic dose. **a** Timeline of experiment in C57Bl6 adults, red lines denote administration of (+)-CBD-oct (20 mg*kg^-1^ p.o.). **b, c** Representative paths of vehicle (*n* = 12) and 20 mg*kg^-1^ ( + )-CBD-oct (*n* = 7) treated animals in the open field. **d** Distance traveled over time (Vehicle: 779.112 ± 29.229; ( + )-CBD-oct: 764.036 ± 38.270). **e** Average speed over time (Vehicle: 2.599 ± 0.0973; ( + )-CBD-oct: 2.544 ± 0.127). **f** Cumulative distance traveled (Vehicle: 9349.342 ± 1509.491; ( + )-CBD-oct: 9168.429 ± 3739.818). **g** Total average speed (Vehicle: 2.599 ± 0.421; ( + )-CBD-oct: 2.544 ± 1.039). All values listed are mean ± standard error, *p* values from Two Way Repeated Measures ANOVA, with Bonferroni post hoc; or t-test. Line graphs are plotted as mean and standard error, and box plots are plotted as median, first and third quartile, and range.

Sedative-hypnotics, including benzodiazepines, remain a first line of treatment for developmental epilepsy syndromes [71]. The sedative effects of benzodiazepines are adverse for children and are thought to contribute to developmental delay [72]. Because (+)-CBD-oct increases δ and θ power in the EEG SAR studies, we screened for potential acutely sedating effects using the open field. Animals were treated with either i.p. saline or (+)-CBD-oct (20 mg*kg^-1^) and placed in the open field (Fig. 3a). ( + )-CBD-oct treated mice show paths of travel that are comparable to vehicle controls (Fig. 3c,c). ( + )-CBD-oct treated mice display a typical habituation curve, with greater distance traveled upon entry, which decreases gradually over time (Fig. 3d). Similar to vehicle controls, ( + )-CBD-oct treated mice also travel at a greater average speed upon entry and decrease speed over time (Fig. 3e). Quantification of cumulative distance and average speed reveal that these measures are not significantly changed compared to vehicle controls (Fig. 3f, g). The lack of acutely sedating effects in the open field, coupled with the SAR effects on EEG δ and θ band power support further examination of (+)-CBD-oct as a promising anti-seizure therapy. The lack of acute sedation further distinguishes (+)-CBD-oct from benzodiazepines and demonstrate its potential for therapeutic development.

### ( + )-CBD-oct controls chemically induced seizures in C57Bl6 adults and in a mouse model of developmental epilepsy syndrome

**Fig. 4:**
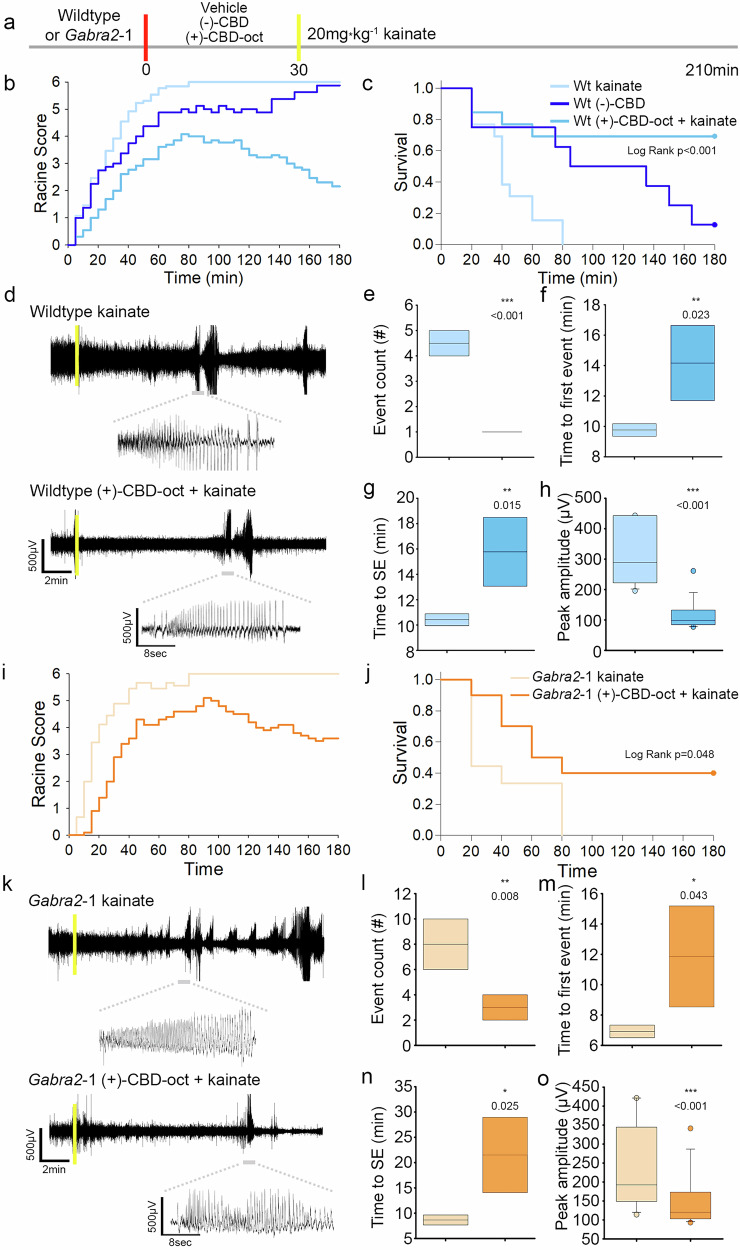
Efficacy of (+)-CBD-oct in control of chemical induced seizure. **a** Timeline of experiment in C57Bl6 adults, red lines denote administration of vehicle or (-)-CBD or (+)-CBD-oct (20 mg*kg^-1^ p.o.), yellow lines denote administration of kainate (20 mg*kg^-1^ i.p.). **b** Quantification of kainate-induced (20 mg*kg^-1^ i.p.) seizure activity according to the Racine scale comparing 20 mg*kg^-1^ (-)-CBD, or (+)-CBD-oct to vehicle treated controls (Vehicle *n* = 13: 5.086 ± 0.409, (-)-CBD *n* = 8, ( + )-CBD-oct *n* = 13: 3.276 ± 0.409). **c** Survival plot of animals following seizure induction (Vehicle *n* = 13: survival time 44.615 ± 5.646, (-)-CBD *n* = 8: survival time 103.750 ± 22.547, ( + )-CBD-oct *n* = 13: survival time 135.385 ± 21.634; Log Rank *p* < 0.001, vehicle vs. (-)-CBD *p* = 0.0133, vehicle vs. (+)-CBD-oct *p* = 0.0018, (-)-CBD vs. (+)-CBD-oct *p* = 0.314). **d** Representative traces of EEG activity in wildtype mice pretreated with vehicle or (+)-CBD-oct following kainate injection (Vehicle *n* = 4, ( + )-CBD-oct n = 4). Yellow line denotes time of kainite administration. **e** Quantification of event count (Vehicle: 4.500 ± 0.577; ( + )-CBD-oct: 1.000 ± 0.000). **f** Time to first seizure event (Vehicle: 9.764 ± 0.476; ( + )-CBD-oct: 14.170 ± 2.853). **g** Time to status epilepticus (SE) (Vehicle: 10.418 ± 0.539; ( + )-CBD-oct: 15.775 ± 3.136). **h** Quantification of peak amplitude following kainate injection (Vehicle: 315.222 ± 98.307.; ( + )-CBD-oct: 124.308 ± 71.588). **i** Quantification of kainate-induced seizure activity according to the Racine scale (*Gabra2*-1 Vehicle *n* = 9: 5.189 ± 0.399, *Gabra2*-1 ( + )-CBD-oct n = 10: 3.576 ± 0.379). **j** Survival plot of animals following seizure induction (*Gabra2*-1 Vehicle *n* = 9: 42.222 ± 9.686, *Gabra2*-1 ( + )-CBD-oct *n* = 10: 102.000 ± 22.652; Log Rank *p* = 0.048). **k** Representative traces of EEG activity in *Gabra2*-1 mice pretreated with vehicle or (+)-CBD-oct following kainate injection. Yellow line denotes time of kainite administration. **l** Quantification of event count (*Gabra2*-1 Vehicle: 8.000 ± 2.309; *Gabra2*-1 ( + )-CBD-oct: 3.000 ± 1.155). **m** Time to first seizure event (*Gabra2*-1 Vehicle: 6.920 ± 0.475; *Gabra2*-1 ( + )-CBD-oct: 11.864 ± 3.837). **n** Time to status epilepticus (SE) (*Gabra2*-1 Vehicle: 8.653 ± 1.130; *Gabra2*-1 ( + )-CBD-oct: 21.508 ± 8.584). **o** Quantification of peak amplitude following kainate injection (*Gabra2*-1 Vehicle: 239.470 ± 111.917; *Gabra2*-1 ( + )-CBD-oct: 154.594 ± 77.503). All values listed are mean (or LS mean) ± standard error, *p* values from Two Way Repeated Measures ANOVA, or Two Way ANOVA, with Bonferroni post hoc; t-test, or Log Rank. Line graphs are plotted as mean, and box plots plotted as median, first and third quartile, and range.

CBD and other cannabimimetics demonstrate protective effects against seizure and seizure-like activity in slice and whole animal [67, 73–75]. We next examined the efficacy of (+)-CBD-oct on modulating kainate-induced seizure in C57Bl6 adults, as well as compared the efficacy of (+)-CBD-oct to (-)-CBD. For these studies, mice were pretreated with either vehicle or (+)-CBD-oct or (-)-CBD (20 mg*kg^-1^; red line) 30 min prior to injection with kainate (20 mg*kg^-1^; yellow line; Fig. 4a). ( + )-CBD-oct pretreated mice show less intense seizures on average, and progress through Racine stages at a decreased rate throughout the testing period compared to mice pretreated with vehicle as well as mice treated with (-)-CBD (Fig. 4b). A survival plot reveals decreased mortality resulting from kainate injection with (+)-CBD-oct pretreatment compared to vehicle and also when compared to (-)-CBD pretreatment (Fig. 4c). (-)-CBD reduced the behavioral presentation of seizures and mortality induced by kainite when compared to vehicle (Fig. 4b, c). To gain detail on how (+)-CBD-oct promotes resilience to the effects of kainite and supports survival, we also examined the effects of (+)-CBD-oct on kainate-induced seizure characteristics in C57Bl6 adults using EEG. Qualitative evaluation of representative EEG traces from C57Bl6 mice treated with 20 20 mg*kg^-1^ kainate (yellow line) suggest that (+)-CBD-oct impacts seizure-like activity compared to vehicle controls (Fig. 4d). Quantification of the number of seizure events in a 20-min period following kainate injection shows a significant decrease in mice pretreated with (+)-CBD-oct compared to vehicle (Fig. 4e). ( + )-CBD-oct pretreatment also increases the latency to the first seizure event (Fig. 4f), along with the time to SE ( < 2 min between seizure events; Fig. 4g). Quantification of peak amplitude of ictal activity reveals that ictal spikes in animals pretreated with (+)-CBD-oct have reduced amplitudes compared to vehicle pretreatment (Fig. 4h).

To extend these studies, we selected a neurodevelopmental disorder model developed and characterized by our lab as prone to spontaneous seizures, with increased sensitivity to chemical seizure induction (*Gabra2*-1) [10, 69]. *Gabra2*-1 mice were selected based on the need to develop more generalizable novel therapies that may be useful in the wide range of treatment-resistant developmental epilepsies. The *Gabra2*-1 model has relevance for the growing number of patients with encephalopathic epilepsy caused by mutation in *GABRA2* [11, 76, 77], as well as *ARHGEF9* intellectual disability syndrome [10, 78], which impairs collybistin and causes a loss of α2 subunit containing GABA_A_ receptors at subtypes of inhibitory synapses [10]. We next characterized the effect of (+)-CBD-oct in altering induced seizure activity in the *Gabra2*-1 mouse. *Gabra2*-1 adults pretreated with (+)-CBD-oct show a reduction in seizure severity compared to vehicle treated *Gabra2*-1 adults as categorized using the Racine scale (Fig. 4i). A survival plot also shows that *Gabra2*-1 mice pretreated with (+)-CBD-oct have an increase in survival compared to vehicle (Fig. 4j). Qualitative EEG traces showing seizure activity in kainite treated (yellow line) *Gabra2*-1 mice indicate alterations in seizure events in (+)-CBD-oct pretreated animals compared to *Gabra2*-1 mice treated with vehicle (Fig. 4k). Quantification of seizure events in the 20-min period following kainate injection show that (+)-CBD-oct pretreated *Gabra2-*1 animals have reduced seizure events (Fig. 4l). ( + )-CBD-oct pretreated *Gabra2-*1 animals also have an increased latency to the first seizure event compared to vehicle (Fig. 4m) accompanied by an increase in time to SE ( < 2 min between seizure events; Fig. 4n). Quantification of peak amplitude of ictal events following kainate treatment is also reduced in (+)-CBD-oct pretreated *Gabra2-*1 animals compared to vehicle (Fig. 4o). These observations demonstrate the utility of (+)-CBD-oct in attenuating the intensity of kainate induced seizures, including efficacy in reducing seizure severity and preventing mortality in a mouse model of developmental epilepsy with increased sensitivity to chemically induced seizure [validated in Supplementary Fig. 3; 69].

### Efficacy of (+)-CBD-oct in normalizing immature spine morphology in a mouse model of developmental epilepsy syndrome

**Fig. 5:**
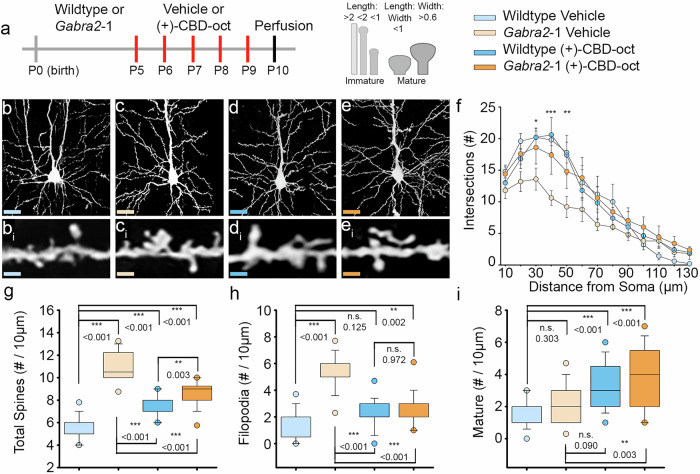
Efficacy of (+)-CBD-oct in normalizing aberrant dendritic arborization, spine density, and morphology in a mouse model of developmental epilepsy. **a** Timeline of developmental administration (red lines) of 20 mg*kg^-1^ ( + )-CBD-oct p.o. or vehicle control; schematic of dendritic spine morphology and classification. **b**–**e** Representative cortical pyramidal cells impregnated with Golgi-Cox stain. **b**_**i**_–**e**_**i**_ Dendritic spine segments (10 µm in length). **f** Sholl analysis of dendritic arbor in pyramidal cells (Wildtype vehicle: 10.631 ± 0.485; *Gabra2*-1 vehicle: 7.369 ± 0.485; Wildtype (+)-CBD-oct: 10.446 ± 0.485; *Gabra2*-1 ( + )-CBD-oct: 10.677 ± 0.485). **g** Total spine density per 10 µm (Wildtype vehicle: 5.651 ± 0.159; *Gabra2*-1 vehicle: 10.941 ± 0.242; Wildtype (+)-CBD-oct: 7.652 ± 0.125; *Gabra2*-1 ( + )-CBD-oct: 8.559 ± 0.203). **h** Quantification of immature filopodia (Wildtype vehicle: 1.440 ± 0.217; *Gabra2*-1 vehicle: 5.320 ± 0.263; Wildtype (+)-CBD-oct: 2.240 ± 0.226; *Gabra2*-1 ( + )-CBD-oct: 2.720 ± 0.255). **i** Quantification of mature dendritic spines (Wildtype vehicle: 1.520 ± 0.165; *Gabra2*-1 vehicle: 2.320 ± 0.256; Wildtype (+)-CBD-oct: 3.320 ± 0.281; *Gabra2*-1 ( + )-CBD-oct: 3.760 ± 0.393). All values listed are mean ± standard error, *p* values from Two Way Repeated Measures ANOVA, or Two Way ANOVA with Bonferroni post hoc; or *t*-test. Line graphs are plotted as mean and standard error, and box plots are plotted as median, first and third quartile, and range.

Benzodiazepines are the primary treatment option for refractory epilepsy in infants and children due to their potentiation of GABA_A_R mediated inhibition. However, chronic benzodiazepine treatment has adverse consequences on dendritic spine dynamics throughout postnatal development [79] and leads to deficits in cognition and behavior [80–82]. Brain region specific, atypical dendritic spine density and morphology is also characteristic of seizure disorders [83]. We next wanted to assess the impact of repeated treatment with (+)-CBD-oct on developing dendritic spines in the *Gabra2*-1 mouse model, with the larger goal of identifying the efficacy of (+)-CBD-oct to attenuate seizure related dendritic spine changes with minimal adverse effects on the developing brain. Wildtype and *Gabra2*-1 pups were given vehicle or (+)-CBD-oct (20 mg*kg^-1^) for 5 consecutive days (P5-P9) before brains were taken for analysis of cell morphology (Fig. 5a). Compared to wildtype pups treated with vehicle, cortical pyramidal cells from *Gabra2*-1 pups display aberrant dendritic arborization and morphology of dendrites (Fig. 5b, c). In particular, *Gabra2*-1 cortical pyramidal cells demonstrate an overall reduction in the arrangement of dendritic processes, measured as a decrease in the number of intersections made by processes radiating from the soma using Sholl analysis. Repeated treatment with (+)-CBD-oct appears to normalize this atypical branching pattern of dendrites, with Sholl analysis of pyramidal cells further confirming that the number of branch intersections radiating from the soma in (+)-CBD-oct treated *Gabra2*-1 pups is comparable to wildtype pups treated with vehicle and (+)-CBD-oct (Fig. 5d–f). Under vehicle conditions, the *Gabra2*-1 mutant also displays atypical dendritic spine density with an abundance of immature filopodia, likely related to the seizure phenotype (Fig. 5c_i_, g, h). Repeated treatment with (+)-CBD-oct in the *Gabra2*-1 mouse reduces the total number of spines (Fig. 5g), specifically decreasing filopodia (Fig. 5h), while promoting mature spines (Fig. 5i). Repeated treatment with (+)-CBD-oct promoted spine maturation in both wildtype and *Gabra2*-1 mice (Fig. 5i). We also examined the impact of repeated (+)-CBD-oct on cell morphology and dendritic spine density in the hippocampus (Supplementary Fig. 4). ( + )-CBD-oct also normalizes atypical dendritic arbor in hippocampal pyramidal cells from *Gabra2*-1 pups to a morphology that is comparable to wildtype animals treated with both vehicle and (+)-CBD-oct (Supplementary Fig. 4b–f). Quantification of total dendritic spines reveals no change in total spine count between wildtype and *Gabra2*-1 pups treated with (+)-CBD-oct, but again (+)-CBD-oct reduces total spines comparing treated and untreated *Gabra2*-1 (Supplementary Fig. 4g). Characterization of dendritic spine morphology shows that the reduction in spine number stems from a loss of immature filopodia in the *Gabra2*-1 mutant (Supplementary Fig. 4h). Mature dendritic spine count remains unchanged in the hippocampus, irrespective of genotype or treatment (Supplementary Figure 4i). Taken together, ( + )-CBD-oct appears to modulate the morphology of the dendritic arbor and spine maturity in both prefrontal cortex and hippocampus. The cellular or circuit level impacts of the dendrite and spine maturation caused by (+)-CBD-oct remain unclear, and future studies should examine functional changes at synapses. These findings highlight the potential of (+)-CBD-oct to promote dendritic and spine maturation in key brain regions during early development, supporting its promise as a disease-modifying treatment for neurodevelopmental epilepsies with minimal disruption to healthy circuit formation.

## Discussion

CBD has emerged as an alternative to benzodiazepines for treating severe, refractory epilepsy in some infants and children. Novel cannabimimetic compounds present opportunities for new therapeutic approaches [65, 84–89]. We show that stereoselective synthesis of the (+)-enantiomer of CBD from (+)-carvone produces derivatives that modulate cortical activity in an alkyl chain length-dependent manner. The derivative with the longest chain, ( + )-CBD-oct, does not reduce open field locomotion, indicating a lack of acute sedative effects. In both chemically induced seizures in C57Bl6 adults and a developmental epilepsy model (*Gabra2*-1), ( + )-CBD-oct reduces seizure frequency and amplitude and prevents mortality. Additionally, it normalizes aberrant spine morphology in *Gabra2*-1 pups without affecting wildtype controls, supporting its potential as a safer, more targeted treatment for pediatric epilepsy. To contextualize these findings, we compared (+)-CBD-oct to the known effects of (-)-CBD across neurophysiological and behavioral domains (Supplementary Table 1).

SAR studies provide insight into the actions of natural (-)-CBD and novel synthetic cannabimimetics. Network level effects of (-)-CBD in humans and rodents demonstrate both broadband and discrete effects on cortical activity. For example, patients with refractory epilepsy who respond to (-)-CBD treatment show a distinct pattern of network integration and synchronicity in δ, θ, and β frequency bands compared to non-responders [90, 91]. In agreement with the varied effects of (-)-CBD on cortical activity, we demonstrate that (+)-carvone-derived (+)-CBD enantiomers modulate EEG broadband activity, but that alkyl chain length may determine specific changes in frequency bands when compared to baseline. The addition of carbon atoms to the alkyl side chain increased both θ and β power following treatment with (+)-CBDP, while an even greater number of carbon atoms on (+)-CBD-oct increased both δ and θ, but not β power. EEG frequency bands reflect distinct patterns of neural network activity, with functional implications for cognition, arousal, and neuropsychiatric conditions. δ oscillations are typically associated with deep sleep and cortical inhibition, but can also emerge in wakefulness during pathological or compensatory network states, such as after seizures or in neurodevelopmental disorders [92]. θ activity, prominent in the hippocampus and medial prefrontal cortex, is linked to memory encoding, navigation, and emotional regulation, and its dysregulation is implicated in both epilepsy and anxiety [93]. Pharmacological modulation of these low-frequency bands has therapeutic potential: enhancing δ may stabilize hyperexcitable networks and promote recovery post-seizure, while increasing θ may normalize hippocampal-prefrontal synchrony associated with anxiety and limbic seizures. Currently characterized antiepileptic therapies influence these bands, with varying behavioral and cognitive outcomes, highlighting their relevance as translational biomarkers in epilepsy and anxiety treatment [94]. Increased β activity differentiates (+)-CBDP and is associated with active wakefulness, motor planning, and cognitive control. In epilepsy, elevated β can reflect compensatory inhibitory processes or, conversely, pathological hypersynchrony, while in anxiety disorders, excessive β has been linked to hypervigilance and cognitive rigidity [95]. Modulating β activity holds translational promise for targeting circuit-level imbalances in both epilepsy and anxiety. The robust yet nuanced effects of the individual (+)-CBD congeners demonstrate that alkyl chain length is a critical factor that could be explored further.

Benzodiazepines have been traditionally used as antiepileptic medications, however, the effects of sedation in children with intractable epilepsy has prompted a need for therapies without adverse sedative effects [96]. To confirm that the changes in δ and θ frequency bands observed following (+)-CBD-oct were not associated with acute sedation, we examined the effect of (+)-CBD-oct on general locomotor behavior in the open field. Diazepam alters locomotor activity, measured by a reduction of mobility time and a decrease in total distance traveled in the open field [97]. In contrast, ( + )-CBD-oct does not reduce measures of locomotion such as distance traveled or average speed. CBD and benzodiazepines differ markedly in their effects on EEG, cognition, and neurobehavioral outcomes despite both enhancing δ oscillations and acting as antiseizure therapies. Benzodiazepines increase δ power via GABA_A_R potentiation and are consistently associated with sedation, cognitive impairment, and motor suppression [94]. While δ oscillations are commonly associated with deep sleep and sedation, they also play roles in attention, motivation, and cognitive processing during wakefulness [98, 99]. For instance, increased δ activity has been linked to internal attention and the inhibition of irrelevant information, suggesting a role in cognitive control [99]. In contrast to benzodiazepines, CBD and its congeners can enhance δ activity without inducing acute sedation, likely reflecting their engagement of distinct targets such as TRP channels, 5-HT₁A receptors, and GPR55. These differences suggest that δ augmentation alone is not sufficient to produce sedation and that CBD-like compounds may promote network stability without disrupting arousal. Additional behavioral assessments, such as the elevated-plus maze and light/dark box, following (+)-CBD-oct may further differentiate the congener’s sedative effects from potential anxiolytic effects [100].

Based on the indication of high potency due to alkyl chain length from the EEG SAR studies and a lack of sedative activity in the open field, we next evaluated the effectiveness of (+)-CBD-oct to attenuate chemically induced seizures. Experimental models of epilepsy using chemical agents include the pilocarpine, pentylenetetrazol, and kainic acid models. The kainic acid model of temporal lobe epilepsy is a well-characterized, reliable model of human SE [101–104]. In rodents and organotypic slices, CBD pretreatment reduces hyperexcitability and susceptibility to subsequent seizures following lithium-pilocarpine and kainic acid [61, 105, 106]. We observe that (+)-CBD-oct exerts anticonvulsant effects on kainate induced seizure. Of particular interest is the reduced severity of the behavioral presentation of chemically induced seizures following pretreatment with (+)-CBD-oct. Additionally, while there was a high incidence of mortality following kainate injection in the mice pretreated with vehicle, ( + )-CBD-oct pretreatment dramatically reduced mortality resulting from kainate. ( + )-CBD-oct also reduced the behavioral signs of seizure and significantly increased survival compared to (-)-CBD. EEG recording revealed that (+)-CBD-oct increased the latency to the first seizure and to SE, as well as decreased the number and amplitude of events compared to vehicle. Overall, ( + )-CBD-oct pretreatment reduces the severity of seizure metrics and further studies are needed to determine the specific molecular, cellular and circuit-level mechanisms through which (+)-CBD-oct is acting to alter seizure characteristics, behavioral presentations, and mortality.

We next wanted to examine the efficacy of (+)-CBD-oct in a context more relevant to developmental epilepsy syndromes in the *Gabra2*-1 mouse model characterized to have spontaneous seizures during development and early mortality, increased susceptibility to kainate-induced seizures, and intellectual disability [10, 69]. Previous studies in rodent models of Angelman syndrome demonstrate that CBD attenuates seizures and EEG interictal activity [107]. These opposing effects on δ oscillations likely reflect model-specific differences in epileptogenic mechanisms and cannabinoid target engagement. In our model of rare developmental epilepsy, δ enhancement by CBD-like compounds may reflect facilitation of physiologic oscillatory activity associated with network stabilization, whereas in other models, suppression of pathologic δ may be more therapeutically beneficial. Additionally, CBD has been shown to attenuate seizure frequency, duration, and severity in a mouse model of Dravet syndrome [67]. In a longitudinal study in adults and children with treatment resistant epilepsy, CBD reduces interictal activity but does not affect other EEG measures of frequency band activity [108]. In the *Gabra2*-1 mouse we found that (+)-CBD-oct pretreatment also attenuates behavioral measures of seizure severity and reduces seizure-related mortality. Quantification of seizure metrics in the EEG also indicate that (+)-CBD-oct pretreatment alters the characteristics of seizure events. As with previous studies examining the effectiveness of (-)-CBD in mouse models of developmental epilepsies [67, 107], ( + )-CBD-oct pretreatment in the *Gabra2*-1 model does not completely abolish seizures arising from kainate. We employed kainate to induce seizures in both wildtype mice and a genetically defined, translationally relevant developmental epilepsy model that exhibits heightened susceptibility to kainate, allowing us to assess treatment effects in a sensitized background. However, reliance on a single chemoconvulsant model constrains generalizability, as it may not capture the full spectrum of seizure mechanisms present in other etiologies or epilepsy types. Future studies could evaluate the differences in (-)-CBD and synthetic (+)-CBD derivatives in terms of mechanisms of reducing seizure severity, and how this may differ across chemical and genetic models.

To further establish the potential of (+)-CBD-oct to be used as a treatment for epilepsy in infants and children, we examined the effects of (+)-CBD-oct on dendrite and spine density and morphology in wildtype and *Gabra2*-1 mice. Benzodiazepines are the first line of treatment for children with refractory epilepsy, however it is well-established that chronic treatment of benzodiazepines is linked to cognitive decline [109] and a reduction of dendritic spine density [79, 110]. We found that 5 consecutive days of treatment with (+)-CBD-oct during early postnatal development did not induce a loss of spines in wildtype pups as might be expected from benzodiazepine treatment. These findings indicate that novel synthetic CBD molecules show promise in treating seizure while minimizing the adverse effects associated with benzodiazepines. Compared to wildtype mice, *Gabra2*-1 animals have increased synaptic density marked by an increase of immature filopodia, which is a characteristic cellular feature of seizure disorders and neurodevelopmental syndromes [83, 111]. We found that 5 consecutive days of treatment with (+)-CBD-oct in *Gabra2*-1 pups normalizes the aberrant dendritic spine phenotype in this model. It is possible that the normalization of these immature spines to mature mushroom dendritic spines is linked to endocannabinoid-promoted neuroplastic changes [112], and further mechanistic studies to examine whether (+)-CBD-oct increases endocannabinoid tone to initiate a homeostatic response to the atypical hyperexcitability identified in rodent models of seizure are needed.

Mechanistically, ( + )-CBD congeners may influence both common and unique targets implicated in the anti-seizure and neuroplasticity effects of (-)-CBD. CBD modulates GABA_A_Rs allosterically, with a preference for α2 subunit containing receptors, which could potentially enhance inhibitory tone without the sedative burden of benzodiazepines [54]. (-)-CBD is a partial agonist and positive allosteric modulator at 5HT_1A_Rs, and its activation hyperpolarizes neurons via GIRK channels and reduces glutamate release [55–57]. (-)-CBD also influences serotonergic tone via 5HT_1A_Rs, with implications for dendritic spine remodeling [113]. (-)-CBD activates and desensitizes TRP channels such as TRPV1, contributing to reductions in hyperexcitability and modulation of synaptic plasticity [63, 114]. GPR55, often described as a pro-convulsant receptor, is antagonized by (-)-CBD, and its inhibition has been associated with normalization of spine morphology and reduced excitatory drive [67, 115]. With particular relevance for (+)-enantiomers, S1P receptors (S1P₁ and S1P₃) have been implicated in neuroinflammatory responses and excitability, with modulation of these pathways influencing seizure outcomes [62, 116–118]. Thus, ( + )-CBD-oct may act through both overlapping and unique mechanisms to modulate both seizure susceptibility and dendritic spine morphology.

This study presents a comprehensive analysis of synthetic carvone-derived CBD enantiomers, highlighting (+)-CBD-oct—the derivative with the longest alkyl chain—as a promising candidate for treating intractable developmental epilepsy. ( + )-CBD-oct significantly reduces seizure-related mortality in both C57Bl6 adults and a developmental epilepsy mouse model, supporting its potential for further preclinical and clinical development. Additionally, its ability to normalize excitatory dendritic spine abnormalities in the epilepsy model underscores the modulatory potential of synthetic (+)-CBD enantiomers and informs the design of novel CBD-based therapies.

## Supplementary information

Supplementary Materials

Supplementary Table 2

## Data Availability

Requests for further information or resources and reagents should be directed to the corresponding author, DJH. The data reported here is stored in an institutional repository, and is available from the corresponding author upon request.
