# Supplementary material for: Carvone derived cannabidiol enantiomers as novel anticonvulsants
Source: Neuropsychopharmacology. 2025 Sep 24;50(13):1970–81. doi: 10.1038/s41386-025-02220-1 (PMC12603161; doi:10.1038/s41386-025-02220-1)
Supplement: Supplementary file 1 — Supplementary Materials [file 41386_2025_2220_MOESM1_ESM.pdf]

# Carvone derived cannabidiol enantiomers as novel anticonvulsants

## Supplementary Material

Rochelle M. Hines[1], April Contreras[1], Adriana Carrillo[1], Alexandra Paton[2], Antonio J. Tenorio[2], William A. Maio[2], Dustin J. Hines[1*]

[1]Department of Psychology, Psychological & Brain Sciences, Interdisciplinary Neuroscience Program, University of Nevada Las Vegas, Las Vegas, NV, USA

[2]Department of Chemistry and Biochemistry, New Mexico State University, Las Cruces, NM, USA

[*]Corresponding Author: dustin.hines@unlv.edu

Keywords: cannabidiol, (+)-enantiomer, developmental epilepsy syndrome, refractory epilepsy

**Supplementary Figure 1.**

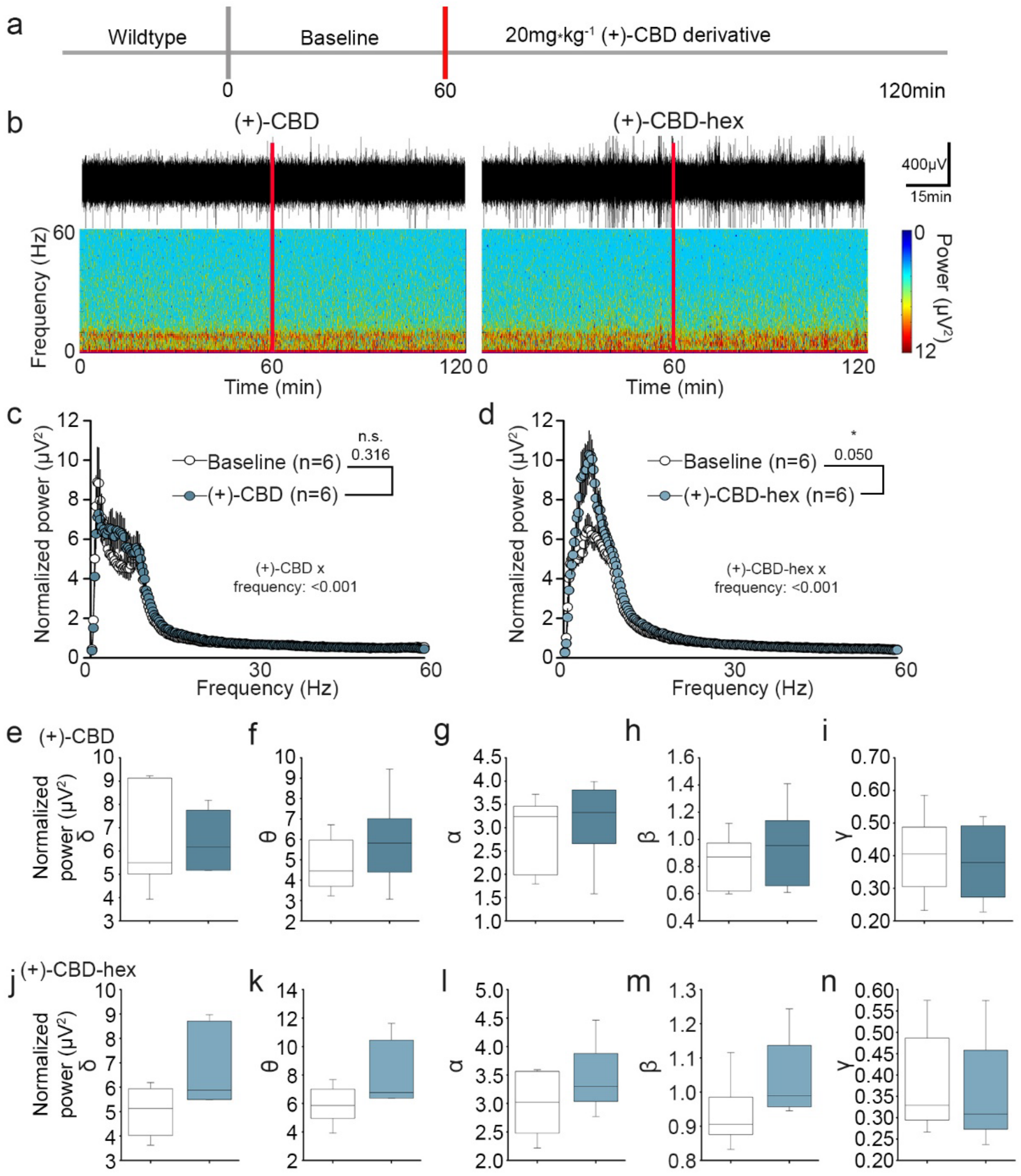


**Structure activity relationship (SAR) study of carvone (+)-CBD derivatives with intermediary alkyl chain lengths. a.** Timeline of experiment, red line denotes administration of (+)-CBD derivatives. **b.** Representative traces (top) and spectrograms (bottom) of EEG recordings following treatment with (+)-CBD derivatives. **c,d.** Fast Fourier transform analysis of the EEG signal comparing (+)-CBD (Baseline: 0.996 ± 0.0365, (+)-CBD: 1.054 ± 0.0365) and (+)-CBD-hex (Baseline: 0.991 ± 0.0467, (+)-CBD-hex: 1.160 ± 0.0467) to vehicle baseline. **e-i.** Spectral analysis of (+)-CBD (All bands n.s.). **j-n.** Spectral analysis of (+)-CBD-hex (All bands n.s). All plots shown and all values listed are mean (or LS mean) ± standard error, *p* values from Two Way Repeated Measures ANOVA with Bonferroni post hoc; or t-test.

**Supplementary Figure 2.**

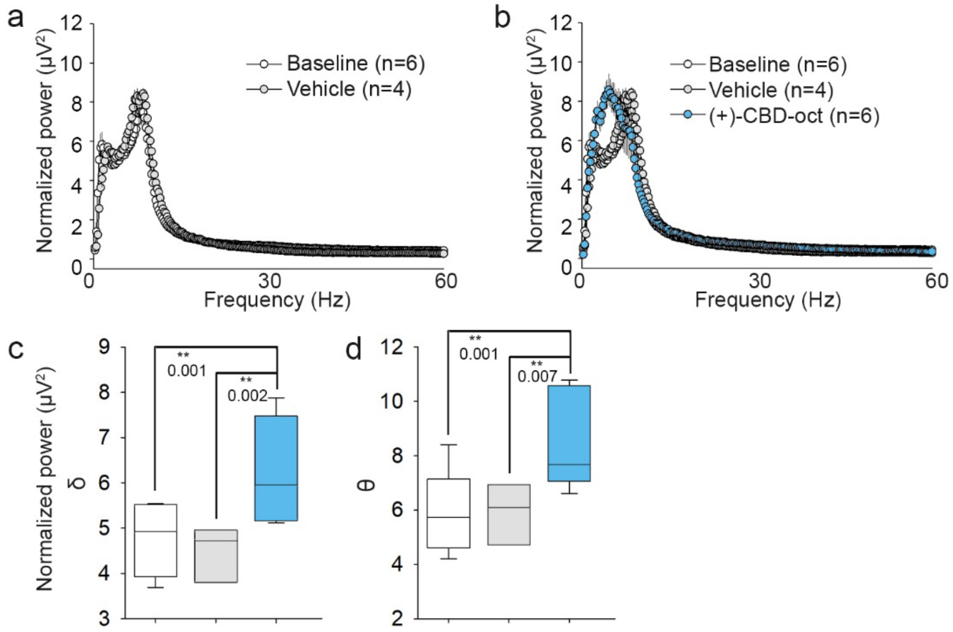


**Baseline control and vehicle control comparison. a.** Fast Fourier transform analysis of the EEG signal comparing Baseline (0.984 ± 0.0210) and Vehicle control (n=4; 0.970 ± 0.0522). **b.** Fast Fourier transform analysis of the EEG signal comparing Baseline (0.984 ± 0.0210) and Vehicle control (0.970 ± 0.0522) to (+)-CBD-oct (1.188 ± 0.0210). **c,d.** Spectral analysis of (+)-CBD-oct compared to Baseline and Vehicle Control for both δ (Baseline: 4.764 ± 0.226; Vehicle control: 4.493 ± 0.222; (+)-CBD-oct: 6.242 ± 0.313) and θ (Baseline: 5.918 ± 0.418; Vehicle control: 5.915 ± 0.407; (+)-CBD-oct: 8.407 ± 0.490). All plots shown and all values listed are mean (or LS mean) ± standard error, *p* values from Two Way Repeated Measures ANOVA, or One Way ANOVA, with Bonferroni post hoc.

**Supplementary Figure 3.**

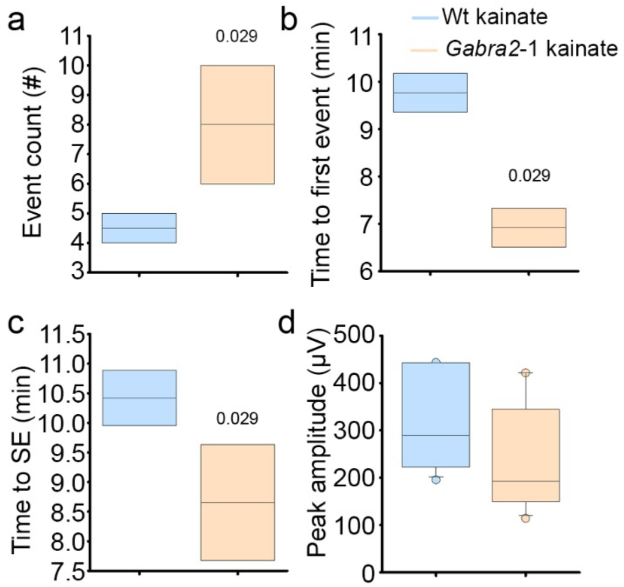


**Seizure severity comparison between wildtype and *Gabra2*-1 mice. a.** Quantification of event count (Wildtype: 4.500 ± 0.577; *Gabra2*-1: 8.000 ± 2.309). **b.** Time to first seizure event (Wildtype: 9.764 ± 0.476; *Gabra2*-1 Vehicle: 6.920 ± 0.475). **c.** Time to status epilepticus (SE) (Wildtype: 10.418 ± 0.539; *Gabra2*-1: 8.653 ± 1.130). **d.** Quantification of peak amplitude following kainate injection (Vehicle: 315.222 ± 98.307.; *Gabra2*-1 Vehicle: 239.470 ± 111.917). All plots shown and all values listed are mean ± standard error, *p* values from t-test.

**Supplementary Figure 4.**

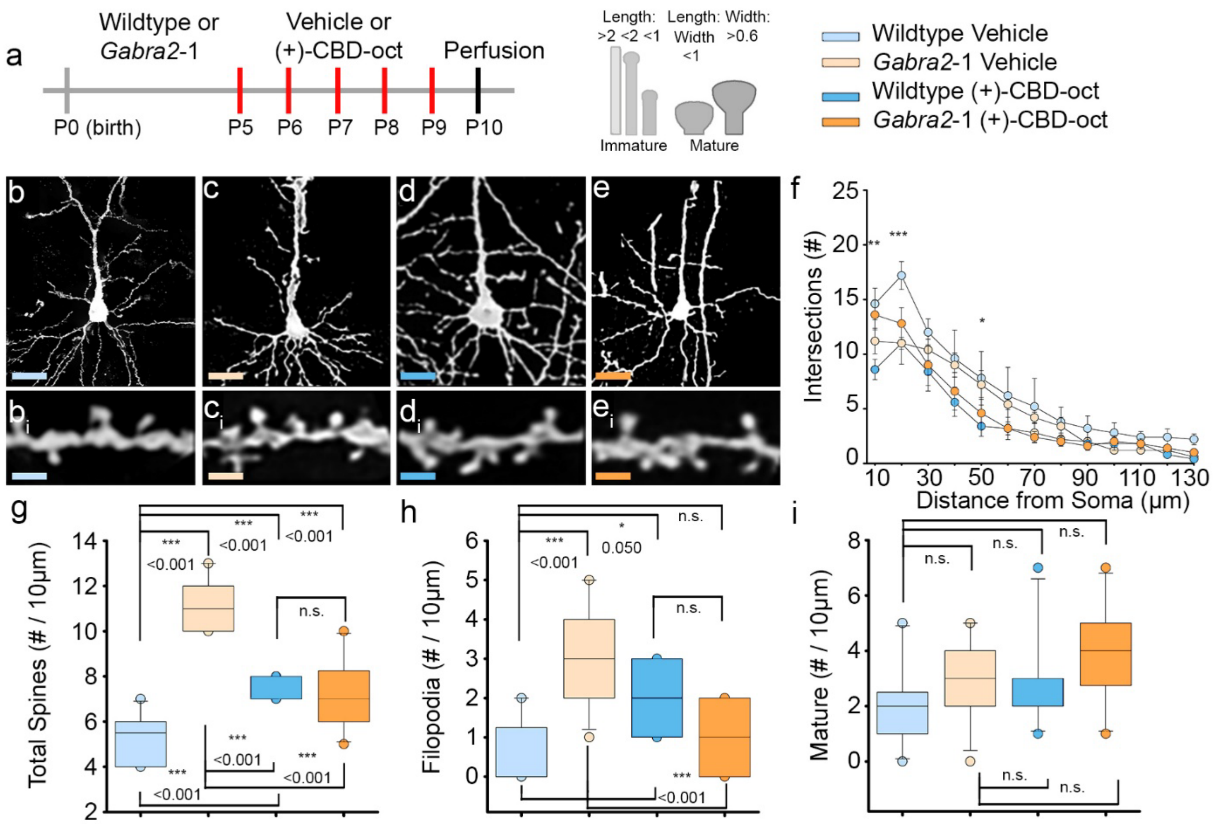


**Effect of (+)-CBD-oct in normalizing aberrant dendritic arborization, spine density, and morphology in the hippocampus.**

**a.** Timeline of experiment, schematic of dendritic spine morphology and classification, and legend. **b-e.** Representative hippocampal pyramidal cells impregnated with Golgi-Cox stain. **$b_i$-$e_i$.** Dendritic spine segments (10µm in length). **f.** Sholl analysis of dendritic arbor in hippocampal pyramidal cells (Wildtype vehicle: 6.877 ± 0.324; *Gabra2*-1 vehicle: 4.154 ± 0.324; Wildtype (+)-CBD-oct: 5.062 ± 0.324; *Gabra2*-1 (+)-CBD-oct: 4.769 ± 0.324). **g.** Total spine density per 10µm (Wildtype vehicle: 5.300 ± 0.335; *Gabra2*-1 vehicle: 11.273 ± 0.333; Wildtype (+)-CBD-oct: 7.700 ± 0.153; *Gabra2*-1 (+)-CBD-oct: 7.300 ± 0.473). **h.** Quantification of immature filopodia (Wildtype vehicle: 0.600 ± 0.267; *Gabra2*-1 vehicle: 3.091 ± 0.415; Wildtype (+)-CBD-oct: 1.900 ± 0.277; *Gabra2*-1 (+)-CBD-oct: 1.000 ± 0.298). **i.** Quantification of mature dendritic spines (Wildtype vehicle: 2.100 ± 0.458; *Gabra2*-1 vehicle: 3.000 ± 0.467; Wildtype (+)-CBD-oct: 2.900 ± 0.504; *Gabra2*-1 (+)-CBD-oct: 3.800 ± 0.533). All plots shown and all values listed are mean (or LS mean) ± standard error, *p* values from Two Way Repeated Measures ANOVA or Two way ANOVA, with Bonferroni post hoc.

**Supplementary Table 1.** Comparison of the established effects of (-)-CBD to (+)-CBD and (+)-CBD-oct.

| **Domain** | **(-)-CBD (~20mgkg$^{-1}$)** | References | **(+)-CBD (20mgkg$^{-1}$)** | **(+)-CBD-oct (20 mgkg$^{-1}$)** |
|---|---|---|---|---|
| EEG – δ Band (0.5–4 Hz) | Mild to moderate increase | Nicholson et al., 2004; Devinsky et al., 2014 | No significant effect | Moderate increase |
| EEG – θ Band (4–8 Hz) | Variable effects; generally neutral to slight increase | Gomes et al., 2011; Campos et al., 2012 | No significant effect | Moderate increase |
| EEG – β Band (13–30 Hz) | Inconsistent; possible slight region dependent increase | Boggs et al., 2018; Laprairie et al., 2015 | No significant effect | No significant effect |
| EEG – γ Band (30–100 Hz) | Inconsistent; possible reduction in hyperexcitable states | Taffe et al., 2015; Silva et al., 2017 | No significant effect | No significant effect |
| Open Field – Mobility (Distance Traveled) | No significant effect | Kaplan et al., 2017; Boggs et al., 2018 | Not tested | No significant effect |
| Seizure Suppression in Animal Models | Effective in MES, PTZ, Scn1a+/− | Devinsky et al., 2014; Kaplan et al., 2017 | Not tested | Effective in Kainate, including in a susceptible model (*Gabra2*-1) |
| Dendritic Arborization | Normalizes reduced dendritic complexity | Kaplan et al., 2017; Silva et al., 2020 | Not tested | Normalizes reduced dendritic complexity |
| Dendritic Spine Morphology | Reduces immature filopodia | Silva et al., 2020; Gobira et al., 2020 | Not tested | Reduces immature filopodia |

**Supplementary Table 2.** Summary of Statistics, see enclosed excel spreadsheet.

## Supplementary Methods

## Library Synthesis

Unless otherwise noted, reactions were performed in flame-dried glassware under an atmosphere of dry nitrogen. Reaction solvents ($CH_2Cl_2$, THF, and $Et_2O$) were purified before use in a Glass Contour Solvent Purification System under a flow of dry nitrogen. All other solvents and reagents were purchased from Sigma-Aldrich and used as received, unless otherwise specified. Thin-layer chromatography (TLC) was performed using plates precoated with silica gel 60 Å F- 254 (250 µm) purchased from Silicycle and visualized by UV light, $KMnO_4$ or anisaldehyde stains, followed by heating. Silicycle SilicaFlash ® P60 silica gel (particle size 40-63 µm) or Silicycle Brand disposable columns were used for flash chromatography. $^1H$ and $^{13}C$ NMR spectra were recorded on a Bruker Advance 500 (operating at 500 MHz and 125 MHz, respectively), and are reported relative to residual solvent peak (δ 7.26 for $^1H$ and δ 77.0 for $^{13}C$ in $CDCl_3$). Data for $^1H$ NMR spectra are reported as follows: chemical shift (δ ppm) [multiplicity, coupling constant (Hz), integration]. Spectra obtained are described using the following abbreviations: s = singlet, d = doublet, t = triplet, q = quartet, m = multiplet. IR spectra were recorded on a Perkin Elmer Spectrum One FTIR Spectrometer and samples were prepared by evaporation from $CHCl_3$ or $CH_2Cl_2$ on NaCl plates. High-resolution mass spectra were obtained through positive electrospray ionization on a Q-Tof Micromass Spectrometer coupled with a Waters Acquity Ultra Performance LC.

***Synthesis of Isopiperitenol:***

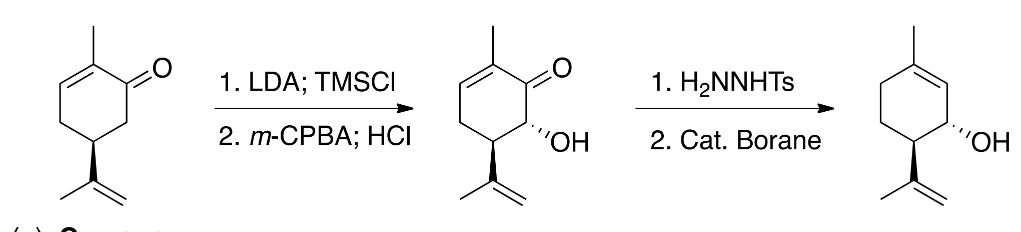


(+)–**Carvone** [(+)-**1**] **SI-1** (+)-**2**

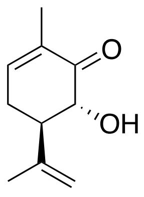

SI-1

6*R*-α-Hydroxy Carvone [**SI-1**]. Was prepared according to the procedure found in *J. Med. Chem*. **2011**, *54*, 3866–3874. $^{1}$H NMR (500 MHz, $CDCl_3$) δ = 6.75 (d, *J* = 6 *Hz*, 1H), 4.94 (s, 1H), 4.92 (s, 1H), 4.15 (d, *J* = 13 *Hz*, 1H), 2.70 (dt, *J* = 5, 11 *Hz*, 1H), 2.51-2.45 (m, 1H), 2.40-2.34 (m, 1H), 1.84 (s, 6H). All other data matches known literature values (*Chem. Eur. J.* **2019** *25* 2983–2988).

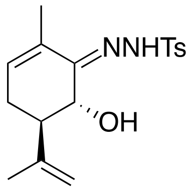

SI-2

Tosylhydrazone **SI-2**. Hydroxycarvone **SI-1** (0.743 g, 4.47 mmol, 1.0 equiv.) was added to a 250 mL round bottom flask and dissolved in $CH_2Cl_2$ (55 mL). To this solution, tosylhydrazide (0.998 g, 5.36 mmol, 1.2 equiv.) was added in a single portion followed by the drop wise addition of acetic acid (0.17 mL, 3.13 mmol, 0.7 equiv.) and concentrated hydrochloric acid (0.1 mL, 4.08 mmol, 0.9 equiv.). The solution was then heated to reflux (40 °C) and allowed to stir for 48h. After the disappearance of starting material was noted *via* TLC analysis, the reaction mixture was quenched by the addition of a 1N aqueous solution of HCl (50 mL) and transferred to a separatory funnel where the aqueous layer was washed with $CH_2Cl_2$ (ca. 3x 50 mL). The combined organic layers were then washed with a saturated aqueous solution of $NaHCO_3$ (ca. 50 mL), followed by brine (ca. 50 mL) before being dried over $MgSO_4$, filtered, and concentrated *in vacuo*. The crude product was purified *via* flash column chromatography (15% EtOAc / 85% Hexanes to 30% EtOAc / 70% Hexanes) to afford the desired product **SI-2** (1.100 g, 74% yield) as a thick yellow foam: $[\alpha]_D^{23}$ = -90.9 (c = 0.4, $CHCl_3$); $^{1}$H NMR (500 MHz, $CDCl_3$) δ = 10.30 (br. s, 1H), 7.84 (d, *J* = 8z *Hz*, 2H), 7.29 (d, *J* = 8 *Hz*), 5.97 (br. d, 1H), 5.02 (s, 1H), 4.91 (s, 1H), 4.44 (d, *J* = 11 *Hz*, 1H), 2.43-2.51 (m, 1H), 2.42 (s, 3H), 2.13-2.17 (m, 2H), 1.79 (s, 3H), 1.72 (s, 3H) ; $^{13}$C NMR (125 MHz, $CDCl_3$) 149.5, 143.6, 143.3, 135.8, 133.1, 131.0, 129.4, 127.9, 115.5, 70.5, 51.1, 28.3, 21.6, 18.8, 18.1 ; IR (neat, thin film) 3470, 3194, 2922, 2857, 1646, 1597, 1450, 1328, 1169, 1030 $cm^{-1}$ ; HRMS (ESI) m/z: $[M+Na]^+$ calc'd for $C_{17}H_{22}O_3N_2SNa$ 357.1248; found 357.1241 m/z.

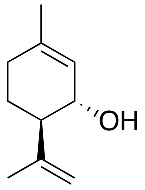

(+)-**2**

1*S*-(–)-Isopiperitenol [**2**]. Tosylhydrazone **SI-2** (0.053 g, 0.158 mmol, 1.0 equiv.) was added to a flame dried 25 mL round bottom flask and dissolved in $CH_2Cl_2$ (3 mL). To this solution at 0 °C was added catecholborane (0.037 mL, 0.347 mmol, 2.2 equiv.) drop wise over the course of 2 min. After the addition was complete, the reaction mixture was allowed to stir for 1 h before NaOAc•$XH_2O$ (0.078 g, 0.573 mmol, 3.6 equiv.) was added in a single portion. After an additional 1 h of stirring, a reflux condenser was attached and the mixture was heated to reflux (40 °C) overnight. After the disappearance of starting material was noted *via* TLC analysis, the reaction mixture was passed over a pad a celite and concentrated *in vacuo*. The crude product was purified *via* flash column chromatography (5% EtOAc / 95% Hexanes to 15% EtOAc / 85% Hexanes) to afford the desired product **2** (0.021 g, 88% yield) as a light yellow oil: $^1$H NMR (500 MHz, $CDCl_3$) δ = 5.44 (s, 1H), 4.89 (s, 1H), 4.85 (s, 1H), 4.11 (d, *J* = 8.5 *Hz*, 1H), 2.05-2.09 (m, 2H), 1.93 (dd, *J* = 4.5, 17.5 *Hz*, 1H), 1.73 (s, 3H), 1.69 (s, 3H), 1.56-1.64 (ddd, *J* = 5.5, 12, 24.5 *Hz*, 2H). All other data matches known literature values (*J. Nat. Prod*. **2018** *81*, 1546–1552).

***Synthesis of CBD analogs:***

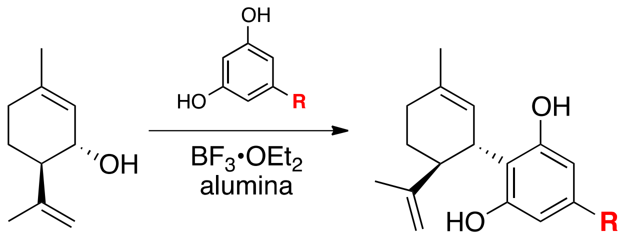


(+)-**2** (+)-**4a–e**

**CBD-2** =

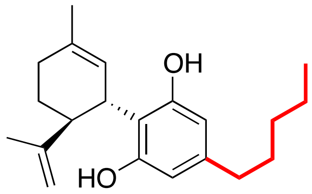

(+)-**4b** [*ent*-(+)-**CBD**]

CBD-2 / (+)-*ent*-CBD [(+)-**4b**] = **(+)-CBD**. Basic alumina (0.920 g, 9.032 mmol, 25.0 equiv.) was added to a flame dried 10 mL roundbottom flask and suspended with $CH_2Cl_2$ (1.5 mL). A reflux condenser was attached to the flask before $BF_3$•$OEt_2$ (0.133 mL, 1.083 mmol, 3.0 equiv.) was added drop wise through the top of the condenser. After being allowed to stir 15 min, the flask was lowered into a pre-heated oil bath and the contents of the flask were boiled for ca. 1 min. To this solution, was added a solution of olivetol (0.078 g, 0.433 mmol, 1.2 equiv.) and 1*S*-(–)-Isopiperitenol [**2**, 0.055 g, 0.361 mmol, 1.0 equiv.) in $CH_2Cl_2$ (1.5 mL) as quickly as possible *via* cannula. After 10 sec, the reaction was quenched by the addition of 2 mL of a saturated aqueous solution of $NaHCO_3$. After cooling to room temperature, the contents of the flask were passed over a pad of celite before being added to a separatory funnel and extracted using $CH_2Cl_2$ (ca. 10 mL). The combined organic layers were then washed with brine (ca. 20 mL) before being dried over $MgSO_4$, filtered, and concentrated *in vacuo*. The crude product was purified *via* flash column chromatography (10% EtOAc / 90% Hexanes to 30% EtOAc / 70% Hexanes) to afford the desired product **4b** (0.0245 g, 22% yield) as an amorphous white solid. Characterization data for (+)-**4b** (known): $^1$H NMR (500 MHz, $CDCl_3$) δ = 6.27 (br. s, 1H), 6.17 (br. s, 1H), 5.97 (br. s, 1H), 5.57 (s, 1H), 4.69 (br. s, 1H), 4.66 (s, 1H), 4.56 (s, 1H), 3.85 (br. d, *J* = 8.5 *Hz*), 2.44 (t, *J* = 8 *Hz*, 2H), 2.40 (dt, *J* = 3, 11 *Hz*, 1H), 2.20-2.26 (m, 1H), 2.07-2.12 (m, 1H), 1.79 (s, 3H), 1.76-1.84 (m, 2H), 1.65 (s, 3H), 1.53-1.59 (m, 2H), 1.25-1.32 (m, 4H), 0.88 (t, *J* = 7 *Hz*, 3H). All other data matches known literature values (*Org. Biomol. Chem.* **2005**, *3*, 1116–1123).

**CBD-1** =

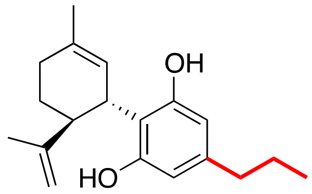

(+)-**4a** [*ent*-(+)-**CBDV**]

CBD-1 / (+)-*ent*-CBDV [(+)-**4a**] = **(+)-CBDV**. Was prepared in a similar manner to (+)-*ent*-CBD [(+)-**4b**] by substituting olivetol with 5-propylbenzene-1,3-diol (0.059 g, 0.394 mmol, 1.2 equiv.). The crude product was purified *via* flash column chromatography (10% EtOAc / 90% Hexanes to 30% EtOAc / 70% Hexanes) to afford the desired product (+)-**4a** (0.0349 g, 37% yield) as an amorphous white solid. Characterization data for (+)-**4a**: $[\alpha]_D^{23}$ = +64.0 (c = 1.9, $CHCl_3$); $^1$H NMR (500 MHz, $CDCl_3$) δ = 6.28 (br. s, 1H), 6.16 (br. s, 1H), 5.99 (br. s, 1H), 5.57 (s, 1H), 4.72 (br. s, 1H), 4.65 (s, 1H), 4.55 (s, 1H), 3.85 (br. d, *J* = 10 *Hz*, 1H), 2.42 (t, *J* = 7.5 *Hz*, 2H), 2.36-2.38 (m, 1H), 2.10-2.26 (m, 1H), 2.07-2.11 (m, 1H),

1.79 (s, 3H), 1.73-1.83 (m, 2H), 1.65 (s, 3H), 1.58 (q, J = 7.5 Hz, 2H), 0.90 (t, *J* = 7 *Hz*, 3H) ; $^{13}$C NMR (125 MHz, $CDCl_3$) δ = 156.2, 154.0, 149.5, 142.8, 140.2, 124.2, 123.8, 113.9, 110.9, 107.7, 46.2, 37.7, 37.3, 30.5, 28.5, 24.1, 23.8, 20.6, 13.9 ; IR (neat, thin film) 3435 (br.), 2865-2965, 1629, 1582, 1448 cm$^{-1}$ ; HRMS (ESI) m/z: $[M+K]^+$ calc'd for $C_{19}H_{26}O_2K$ 325.2916; found 325.1598 m/z.

**CBD-4** =

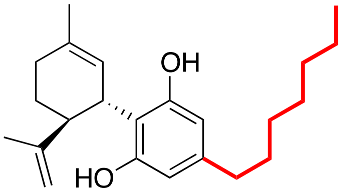


(+)-**4d** [*ent*-(+)-**CBDP**]

CBD-4 / (+)-*ent*-CBDP [(+)-**4d**] = **(+)-CBDP**. Was prepared in a similar manner to (+)-*ent*-CBD [(+)-**4a**] by substituting olivetol with 5-heptylbenzene-1,3-diol (0.0782 g, 0.375 mmol, 1.2 equiv.). The crude product was purified *via* flash column chromatography (10% EtOAc / 90% Hexanes to 30% EtOAc / 70% Hexanes) to afford the desired product (+)-**4d** (0.0232 g, 22% yield) as an amorphous white solid. Characterization data for (+)-**4b**: $[\alpha]_D^{23}$ = +126.0 (c = 0.02, MeCN); $^1$H NMR (500 MHz, $CDCl_3$) δ = 6.28 (br. s, 1H), 6.16 (br. s, 1H), 5.97 (br. s, 1H), 5.56 (s, 1H), 4.66 (s, 1H), 4.59 (br. s, 1H), 4.55 (s, 1H), 3.84 (br. d, *J* = 8.5 *Hz*, 1H), 2.43 (t, *J* = 8 *Hz*, 2H), 2.39 (dt, *J* = 5, 10.5 *Hz*, 1H), 2.19-2.26 (m, 1H), 2.06-2.12 (m, 1H), 1.79 (s, 3H), 1.72-1.83 (m, 2H), 1.65 (s, 3H), 1.52-1.57 (m, 3H), 1.24-1.29 (m, 7H), 0.87 (t, *J* = 7 *Hz*, 3H) ; $^{13}$C NMR (125 MHz, $CDCl_3$) δ = 156.2, 153.9, 149.5, 143.1, 140.2, 124.2, 113.8, 110.9, 109.9, 108.1, 46.2, 37.4, 35.6, 31.9, 31.1, 30.5, 29.4, 29.3, 28.5, 23.8, 22.8, 20.6, 14.2 ; IR (neat, thin film) 3433 (br.), 2855-2958, 1629, 1584, 1443 cm$^{-1}$ ; HRMS (ESI) m/z: $[M]^+$ calc'd for $C_{23}H_{35}O_2$ 343.2639; found 343.2639 m/z.

**CBD-3** =

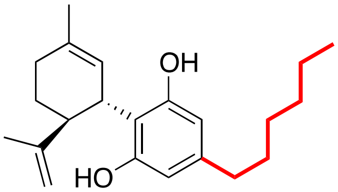


(+)-**4c** [*ent*-(+)-**CBD-Hex**]

CBD-3 / (+)-*ent*-CBD-Hex [(+)-**4c**] = **(+)-CBD-hex**. Was prepared in a similar manner to (+)-*ent*-CBD [(+)-**4a**] by substituting olivetol with 5-hexylbenzene-1,3-diol (0.061 g, 0.313 mmol, 1.0 equiv.). The crude product was purified *via* flash column chromatography (10% EtOAc / 90% Hexanes to 30% EtOAc / 70% Hexanes) to afford the desired product (+)-**4c**

(0.036 g, 35% yield) as an amorphous white solid. Characterization data for (+)-**4c**: $[\alpha]_D^{23}$ = +66.86 (c = 0.3, $CHCl_3$); $^1H$ NMR (500 MHz, $CDCl_3$) δ = 6.28 (br. s, 1H), 6.16 (br. s, 1H), 5.98 (br. s, 1H), 5.57 (s, 1H), 4.69 (br. s, 1H), 4.66 (s, 1H), 4.55 (s, 1H), 3.85 (br. d, *J* = 10 *Hz*, 1H), 2.43 (t, *J* = 8 *Hz*, 2H), 2.36-2.40 (m, 1H), 2.07-2.26 (m, 2H), 1.79 (s, 3H), 1.71-1.82 (m, 2H), 1.65 (s, 3H), 1.53-1.57 (m, 2H), 1.25-1.30 (m, 6H), 0.86-0.89 (m, 3H), ; $^{13}C$ NMR (125 MHz, $CDCl_3$) δ = 154.7, 154.1, 149.4, 143.0, 140.0, 124.1, 113.7, 110.8, 110.0, 107.5, 46.1, 37.2, 35.5, 31.7, 30.9, 30.4, 28.9, 28.4, 23.7, 22.6, 20.5, 14.1 ; IR (neat, thin film) 3444 (br.), 2853-2961, 1629, 1582, 1446, 1028 $cm^{-1}$ ; HRMS (ESI) m/z: $[M+Na]^+$ calc'd for $C_{22}H_{32}O_2Na$ 351.2288 m/z .

**CBD-8** =

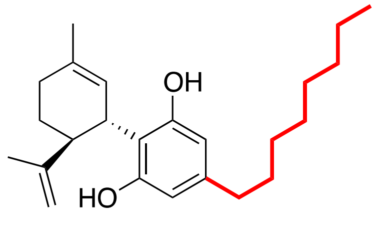


(+)-**4e** [*ent*-(+)-**CBD-oct**]

CBD-8 / (+)-*ent*-CBD-Oct [(+)-**4e**] = **(+)-CBD-oct**. Was prepared in a similar manner to (+)-*ent*-CBD [(+)-**4a**] by substituting olivetol with 5-octylbenzene-1,3-diol (0.086 g, 0.394 mmol, 1.2 equiv.). The crude product was purified *via* flash column chromatography (10% EtOAc / 90% Hexanes to 30% EtOAc / 70% Hexanes) to afford the desired product (+)-**4e** (0.0324 g, 28% yield) as an amorphous white solid. Characterization data for (+)-**4e**: $[\alpha]_D^{23}$ = +71.64 (c = 0.1, $CHCl_3$); $^1H$ NMR (500 MHz, $CDCl_3$) δ = 6.27 (br. s, 1H), 6.15 (br. s, 1H), 5.97 (br. s, 1H), 5.56 (s, 1H), 4.65 (s, 1H), 4.55 (s, 1H), 3.84 (br. d, *J* = 8.5 *Hz*, 1H), 2.43 (t, *J* = 8 *Hz*, 2H), 2.39 (dt, *J* = 2.5, 11 *Hz*, 1H), 2.20-2.25 (m, 1H), 2.06-2.12 (m, 1H), 1.79 (s, 3H), 1.75-1.83 (m, 2H), 1.65 (s, 3H), 1.20-1.60 (m, 12H), 0.87 (t, *J* = 6.5 *Hz*, 3H) ; $^{13}C$ NMR (125 MHz, $CDCl_3$) δ = 156.2, 154.0, 149.5, 143.1, 140.2, 124.2, 113.8, 100.9, 109.9, 108.0, 46.2, 37.4, 35.6, 32.0, 31.1, 30.5, 29.6, 29.4, 29.4, 28.5, 23.8, 22.8, 20.6, 14.2 ; IR (neat, thin film) 3443 (br.), 2855-2955, 1628, 1581, 1445 $cm^{-1}$ ; HRMS (ESI) m/z: $[M]^+$ calc'd for $C_{24}H_{37}O_2$ 357.2795; found 357.2785 m/z.
